# Quiescent Hepatic Stellate Cells Functionally Contribute to the Hepatic Innate Immune Response via TLR3

**DOI:** 10.1371/journal.pone.0083391

**Published:** 2014-01-08

**Authors:** Caroline L. Wilson, Jelena Mann, Meagan Walsh, Maria J. Perrugoria, Fiona Oakley, Matthew C. Wright, Chiara Brignole, Daniela Di Paolo, Patrizia Perri, Mirco Ponzoni, Michael Karin, Derek A. Mann

**Affiliations:** 1 Institute of Cellular Medicine, Faculty of Medical Sciences, Newcastle University, Newcastle upon Tyne, United Kingdom; 2 Experimental Therapy Unit, Laboratory of Oncology, Istituto Giannina Gaslini, Genoa, Italy; 3 Laboratory of Gene Regulation and Signal Transduction, Department of Pharmacology and Pathology, School of Medicine, University of California San Diego, La Jolla, California, United States of America; Centre d'Immunologie de Marseille-Luminy, CNRS-Inserm, France

## Abstract

Toll-like Receptor 3 (TLR3) is a pathogen pattern recognition receptor that plays a key role in innate immunity. TLR3 signalling has numerous functions in liver, both in health and disease. Here we report that TLR3 is expressed by quiescent hepatic stellate cells (HSC) where it functions to induce transcription and secretion of functional interferons as well as a number of other cytokines and chemokines. Upon transdifferentiation into myofibroblasts, HSCs rapidly loose the ability to produce interferon gamma (IFNγ). Mechanistically, this gene silencing may be due to Polycomb complex mediated repression via methylation of histone H3 lysine 27. In contrast to wild type, quiescent HSC isolated from *tlr3* knockout mice do not produce IFNγ in response to Poly(I∶C) treatment. Therefore, quiescent HSC may contribute to induction of the hepatic innate immune system in response to injury or infection.

## Introduction

Hepatic stellate cells (HSC) are specialised pericytes of the liver sinusoids found in the Space of Dissè, a basement membrane-like structure located between columns of hepatocytes and sinusoidal endothelial cells [1]. The most characterised function of HSCs is the ability to transdifferentiate from a quiescent phenotype into highly proliferative, contractile and wound-healing myofibroblast [2]. This so-called activated HSC (aHSC) produces vast quantities of fibril-forming collagens and promotes net deposition of fibrotic extracellular matrix, a process important for repair following infection or trauma to the liver. However, in the chronically injured liver, this normal physiological process may become dysregulated and lead to the development of fibrosis [1].

The role of quiescent HSCs (qHSC) has received much less attention. A known key function is storage of Vitamin A which is found in numerous intracellular droplets [3], [4]. Additionally, several investigators have described that qHSC possess multiple thorn-like cytoplasmic extensions which can protrude into the sinusoidal space or make direct contact with hepatocytes. These membrane projections have been shown to function as a leading edge for the qHSC and play a role in sensing of extracellular factors that influence HSC phenotype. Given the anatomical location of qHSCs and their morphology, they have the potential to operate as sinusoidal sentinels, detecting mechanical or biochemical alterations in hepatocytes, endothelial cells, within the Space of Dissè or even within the sinusoidal spaces [4].

The innate immune system serves as a “first defence” responding to acute tissue trauma or infection by mounting protective anti-microbial and wound-healing response. These responses are mediated by a variety of immune cells including recruited neutrophils, mast cells, eosinophils, natural killer (NK) cells and tissue macrophages. The molecular triggers for innate immunity are pattern recognition receptors (PRRs) [5] including the IL-1 Receptors [6] (reviewed in [7]), members of the Toll-like Receptor (TLR) family [8] and Nucleotide Oligomerisation Domain (NOD)-like receptor families [9]. A total of 13 distinct mammalian TLRs (TLR1-13) have been identified to date and each responds to specific ligands of microbial origin or from the intracellular contents of damaged or dying host cells. Upon engagement by their ligands, the TLRs trigger a cascade of intracellular signalling pathways that culminate in induction of genes encoding interferons, cytokines and chemokines required for the recruitment and activation of innate immune cells [10].

Activated human HSCs express TLR4 and are able to respond to its ligand lipopolysaccharide (LPS) with activation of IKK/NF-κB and subsequent expression of a variety of pro-inflammatory cytokines [11]. Mouse HSCs are reported to express TLR2, 4 and 9 and are responsive to their respective ligands which induce the secretion of IL-6, TGFβ1 and MCP1 [12]–[14]. Recent studies by Seki *et al*, have described how TLR4 on activated mouse HSC is required for sensitising the cells to TGFβ1, specifically by promoting down-regulation of the TGFβ1 pseudo-receptor Bambi [13]. The observation that mice lacking TLR4 or its downstream adaptor Myd88 were resistant to fibrosis induced by a variety of different injury mechanisms highlighted the importance of innate immune receptor in hepatic wound-healing response.

In the present study, we have addressed the question of which TLRs are expressed and functional on qHSC. We focused on TLR3, a sensor of dsRNA, which is expressed at relatively high levels on both qHSC and aHSC but has distinct functions in these two phenotypic states. Our data suggest that engagement of TLR3 on qHSC results in induction of interferons (α,β and γ) and cytokine (IL-6) gene expression. However, aHSC lose the capacity to induce interferons in response to TLR3, but retain TLR3-induction of IL-6. Our data suggest that qHSC may play a significant contributing role to TLR3-mediated interferon-dependent innate immune responses.

## Materials and Methods

### Ethics statement

All animal experiments were undertaken in accordance with appropriate licences for animal experiments which were issued/approved by local ethical committee and UK Home Office.

### Animals and models of liver disease

#### Chronic CCl_4_ liver injury model

Fibrogenesis was induced by 4-week CCl_4_ treatment of 250 g adult Sprague-Dawley rats. Rats were injected intraperitoneally (IP) twice weekly with CCl_4_/olive oil mix in a 1∶3vol/vol ratio at 2 µl/g body weight. Twenty-four hours after the final CCl_4_ administration, animals were sacrificed and blood and tissues harvested.

#### Acute CCl_4_ liver injury

Single dose of CCl_4_ was given by IP injection prepared as CCl_4_/olive oil in a 1∶1vol/vol ratio at 2 µl/g body weight to both rats and mice. Animals were sacrificed at varying times after injury (as stated in figures and figure legends) and tissues harvested for histological and molecular analysis.

### SDS-PAGE and Immunoblotting

SDS-PAGE and immunoblotting was performed as previously described [15]. Primary antibodies recognising TLR3 (AnaSpec), IRAK1, TRAF6 (Santa Cruz) were used at 1∶1000 dilution and GAPDH (Abcam) at 1∶2000. Membranes were probed with the appropriate secondary antibody (anti-mouse Sigma; anti-rabbit Cell Signalling Technologies) and proteins visualised using chemiluminescence (Pierce).

### Immunocytochemistry

Formalin fixed, paraffin embedded tissue was dewaxed and rehydrated in decreasing concentrations of ethanol. Sections underwent antigen retrieval in citric saline solution and were subsequently permeabilized with 0.1% saponin in 0.5% bovine serum albumin (BSA). Toll-like receptor 3 primary antibody (AnaSpec) was used in a concentration of 1∶1000 and incubated at room temperature for 1 hour. Coverslips were mounted in DAPI-containing fluorescent mounting media.

### Quantitative Reverse Transcriptase-Polymerase Chain Reaction (qRT-PCR)

Total RNA was purified from isolated cells or whole liver using the RNeasy Mini Kit (Qiagen, UK). cDNA was generated using random hexamer primers and MMLV reverse transcriptase enzyme (Promega, UK). Quantitative PCR was performed on an ABI 7500 with a 3 step amplification program: 20 sec at 94°C, 40 cycles of 20 sec at 55°C, 30 sec at 72°C and 5 sec at 94°C. All reactions were normalised to GAPDH and relative level of transcriptional difference calculated using the following equation: 1/(2A)×100. (Primer sequences are listed in Table 1).

**Table 1:** Table of primers.

Species	Gene	Forward	Reverse
Rat	Collagen	ttcacctacagcacgcttgtg	gatgactgtcttgccccaagt
Rat	β-actin	agccatgtacgtagcccatcc	ctccagctgtggtggtgaa
Rat	α-SMA	cgaagcgcagagcaagaga	catgtcgtcccagttggtgat
Rat	IL-1β	ttcaaatctcacagcagcat	catcccacgagtcacagagg
Rat	IL-6	acaagataacaagaaagacaaa	Agtcttttatctcttgtttga
Rat	Interferon α	ggctcggctctgtgctttct	atttgtgccaggagtgtgaa
Rat	Interferon β	actgggtggaatgagactat	taaagtagtcgtggatgtca
Rat	Interferon γ	ggatgctatggaaggaaaga	gcgattcgatgacacttatg
Rat	CCL2/MCP1	ggaccagaaccaagtgagatc	gaggtggttgtggaaaagaga
Rat	CCL5/RANTES	catccctcaccgtcatcctc	tctgggttggcacacacttg
Rat	TNF-α	gccaccacgctcttctgtct	cctctgcttggtggtttgctac
Rat	MxA	actccatcctgcaaacatttgggc	accagttgcacttactggtgtcct
Rat	TIMP1	gcaactcggacctggtcataa	cggcccgtgatgagaaact
Rat	TLR1	tccagtatcttaatatcagtg	catataggcagggcatcaaa
Rat	TLR2	tgtcagtggccagaaaagatg	agattgttgttactaacatc
Rat	TLR3	agccttcaacgactgatgct	atttctagattctcaagacc
Rat	TLR4	gtaaagaatttagaagaagg	gagcaatctcatattcaaag
Rat	TLR5	cctgctcagcttcaactata	ctaagattgggcaggtttct
Rat	TLR6	taatattaaattgaatgatga	gttaagttgtaaatattgag
Rat	TLR7	aaaactgttattatcgaaat	gctgtgacattgttatct
Rat	TLR8	tagaggagagggattggg	tcatccattagcctctgcaa
Rat	TLR9	tcaatggctctcagttcctg	aagggctggctgttgtagct
Rat	TLR10	tggcaagagccagtttgt	cccagagcaggtcaactttat
Rat	TLR11	cctttcctcctacatcccattc	cctctgtatttctgggcactt
Rat	TLR12	ctgtgtctactctgcttcc	aaggcatcaggaggtaga
Rat	TLR13 - like	cagaggccattagtgacatacc	ccagagcagacagattagtgaaa

### Reagents

#### TLR ligands

Rat and mouse HSCs were incubated with TLR ligands (InvivoGen) as detailed in figure legends. The ligands and their concentration in cell culture were (unless otherwise stated) - TLR2 (Lipoteichoic (LTA), 100 ng/ml), TLR3 (Poly (I∶C), 1 µg/ml), TLR4 (lipopolysaccharide (LPS), 100 ng/ml), TLR5 (flagellin, 1 µg/ml), TLR7/8 (Imiquimod, 1 µg/ml) and TLR9 (stimulatory CpG ODN, 10 µg/ml). IL-1α was used at a concentration of 2 ng/ml (Peptroech 211-11A), IFNγ at 100 ng/ml (Peprotech 315-05). Transcriptional inhibitors, actinomycin D and 5,6-Dichlorobenzimidazole Riboside (DRB) were purchased from Sigma Aldrich (A9415 and D1916 respectively). The C13-GT was used at a concentration of 20 µg/gram of body weight. Clodronate-liposomes were a kind gift from Professor Mirco Ponzoni and injected intraperitoneally at a concentration of 25 µg/gram of body weight.

### *tlr^−/−^3* knockout mice

The knockout mice were obtained from Dr Lars Eckmann, University of Southern California, San Diego, USA. Authors wish to thank Dr Eckmann for his help and support with breeding and supplying the mice. The knockout mice were originally generated as described in [16].

### Hepatic Stellate Cell isolation

Rat and mouse HSCs were isolated from 250 g male Sprague-Dawley rats and 25–30 g adult male mice respectively, by sequential perfusion with collagenase B (Roche) and pronase (Roche) and quiescent HSCs separated by discontinuous density centrifugation in 11.5% Optiprep (Sigma Aldrich D1556). Mouse and rat primary HSCs were maintained at 37°C (5% CO_2_) in Dulbecco's Modified Eagle's Media supplemented with 16% foetal bovine serum (FBS), 100 U/ml penicillin, 100 µg/ml streptomycin, 2 mM L-glutamine (Life Technologies). Culturing of freshly isolated quiescent HSC on tissue culture plastic leads to their activation over a period of 7 to 10 days and spontaneous acquisition of a myofibroblast phenotype, which is thought to be highly representative of *in vivo* HSC activation.

### Murine Macrophage isolation

Bone marrow cells were isolated from the femurs of C57/Blk6 mice as previously described [17]. Briefly, cells were differentiated into macrophages by culturing for 7 days with media supplemented with 5% horse serum (Sigma Aldrich H1270) and 10% of L929 cell line conditioned media (that contains M-CSF).

### Enzyme linked Immunosorbent Assay

Culture supernatant concentration of IL-6 was measured using a rat IL-6 Quantikine ELISA according to manufacturer's instructions (R&D Systems Minneapolis, MN).

### Fluorescence activated cell sorting (FACS)

Cells were incubated at 4°C for 1 hr with 24G2 antibody that prevents non-specific Fc receptor binding, followed by 1 hr incubation at 4°C with anti MHC class II-FITC conjugated antibody (eBioscience 11-0920-82). Following incubation, cells were washed and resuspended in 2% FBS in phosphate-buffered saline. Up to 10,000 events were analysed on FACScan/FACS Canto II (BD, Oxford, UK) using Flowjo software (FlowJo, Inc).

### Chromatin Immunoprecipitation Assay

Antibodies used for immunoprecipitation were purchased from: histone H3 di methyl K27 (H3K27me2, Abcam) and Histone H3 trimethyl K27 (H3K27me3, Diagenode). 10 µg of each antibody or appropriate irrelevant antibody control were used in each ChIP reaction as described previously [18]. Primers used for detection of relevant rat genomic sequences were: IFNγ −43 kb sense 5′- aaggtcaagccataacattc-3′ and antisense 5′- cagggatgaacaaggaccag-3′; IFNγ −0.5 kb sense 5′- cttttgtaaccgaacgccttc-3′ and antisense 5′- cttttacttcacaccatttg-3′; IFNγ 0.4 kb sense 5′- tcggtgaggtgttcgttgac-3′ and antisense 5′- aagaatgaaaaccatgaagg-3′ and IFNγ 1.1 kb sense 5′- gagttgagtttatttgtgg-3′ and antisense 5′- ctgtggagttttgttgaatg-3′. Each PCR reaction was performed in triplicate and the analysis was repeated three times from independent ChIP experiments. A signal intensity value for each sample was calculated from the average of the experiments. Average values of eluates were normalized to average values of control antibody sample and expressed as fold enrichment above background (i.e. control antibody) [18].

### Statistical Analysis

Data are expressed as means ± standard error of the mean (SEM). All P values were calculated using a two-tailed paired or unpaired Student t test. Statistically significant data is represented in figures where *, **, and *** denote P values of <0.05, <0.01 and <0.001, respectively.

## Results

### TLR3 is expressed and functional in quiescent and activated HSC

**Figure 1:**
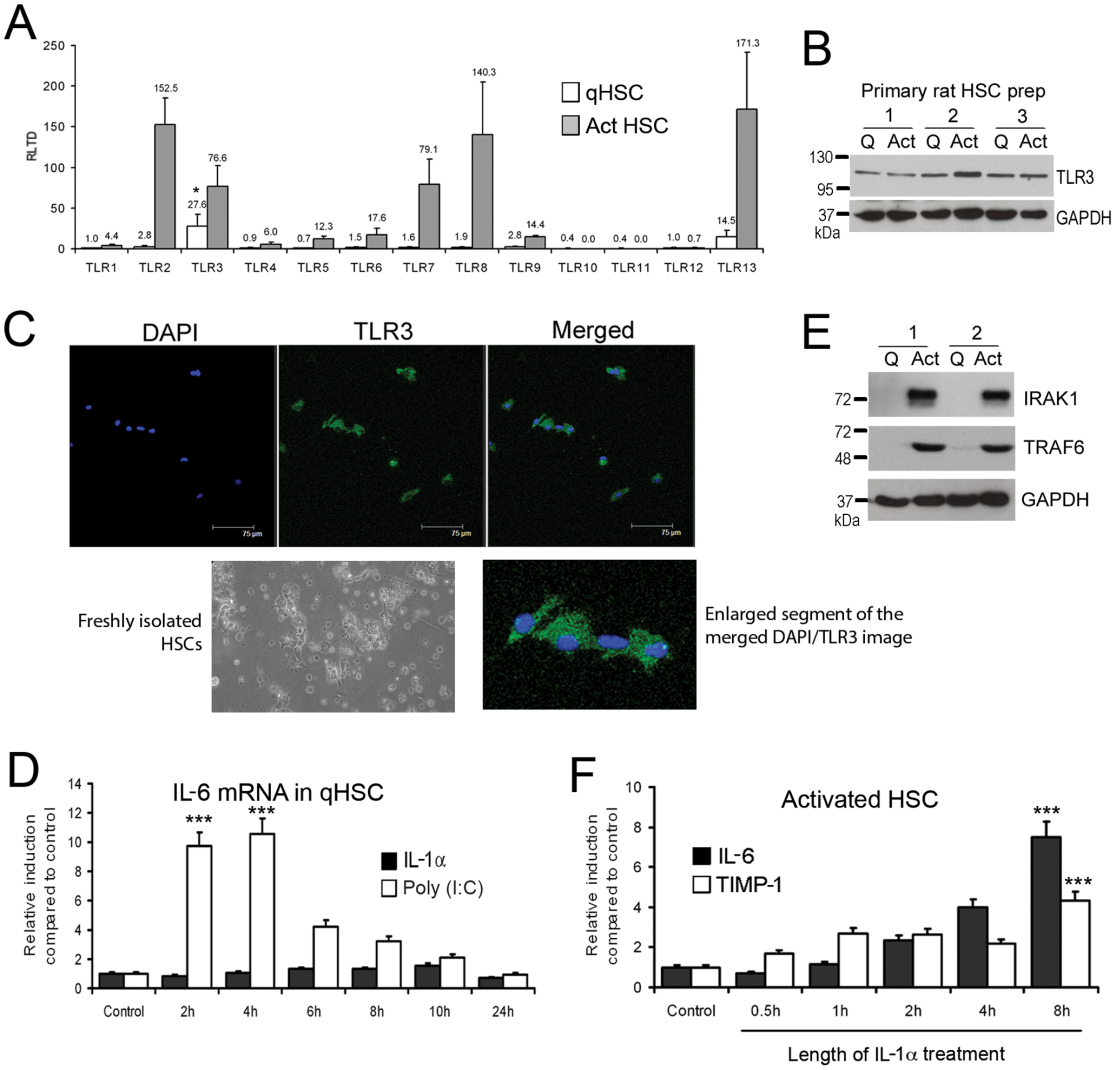
Quiescent and activated HSCs express Toll like receptors. (A) mRNA levels of TLR1-13 were quantified by qRT-PCR in three separate preparations of primary rat qHSCs (day 0) and day 10 transdifferentiated myofibroblasts. Data are expressed as relative level of transcriptional difference (RLTD) to TLR1 mRNA expression (n = 3). (B) Thirty micrograms of whole cell protein extract from three separate preparations of quiescent rat HSCs (culture day 1) or activated myofibroblasts (culture day 10) were separated by SDS-PAGE and immunoblotted for TLR3 and GAPDH. (C) Toll-like receptor 3 was visualised in the cytoplasm of rat qHSCs (*ex vivo*) culture day 1 (bar represents 75 µm). (D) Quiescent rat HSCs were treated with Poly(I∶C) (1 µg/ml) or IL-1α (2 ng/ml) for up to 24 hours; IL-6 mRNA was measured and normalised to β actin (n = 3). (E) Thirty micrograms of whole cell protein extract from two separate preparations of quiescent HSCs or activated myofibroblasts (culture day 10) were separated by SDS-PAGE and immunoblotted for IRAK1, TRAF6 and GAPDH. (F) Activated rat myofibroblasts were treated with IL-1α (2 ng/ml) for up to 24 hours; IL-6 and TIMP1 mRNA were measured and normalised to β actin (n = 3). (*p<0.05, ***p<0.001).

To determine relative expression of TLRs between qHSC and aHSC we measured transcript expression for TLR1-13 in freshly isolated or culture-activated rat HSC. TLR2, 3, 7, 8 and 13 transcripts were all induced with culture activation and were expressed at relatively high levels compared with other TLRs (Figure 1A). The highest expressed TLR transcripts in qHSC were TLR3 (27.57±15.16 RLTD) and TLR13 (14.54±8.09 RLTD) (p<0.05 compared with other TLR transcripts). Western blot analysis confirmed that TLR3 protein is expressed in both qHSC and aHSC, however no significant change in expression occurred with activation (Figure 1B). Furthermore, we confirmed presence of TLR3 in qHSC (culture day 1) by immunocytochemistry (Figure 1C). As no previous studies have reported a role for TLR3 in qHSC we determined if they are responsive to the TLR3 ligand Poly(I∶C). As illustrated in Figure 1D, qHSCs treated with Poly(I∶C) underwent a transient increase in IL-6 mRNA transcript that peaked between 2 (9.7 RLTD, p<0.001) and 4 (10.54 RLTD, p<0.001) hours following treatment (Figure 1D). By contrast qHSC were unresponsive to IL-1α (Figure 1D) which may be explained by absence of key downstream signalling factors TRAF6 and IRAK1 in quiescent HSC, which are subsequently induced during HSC activation (Figure 1E). As expected, aHSC were fully responsive to IL-1α treatment which stimulated the expression of IL-6 and TIMP-1 indicating a profibrogenic phenotype (Figure 1F). Maximal induction of IL-6 and TIMP-1 occurred after 8 hours of IL-1α treatment (7.69±1.14 RLTD, 4.3±0.62 RLTD, respectively p<0.001)

**Figure 2:**
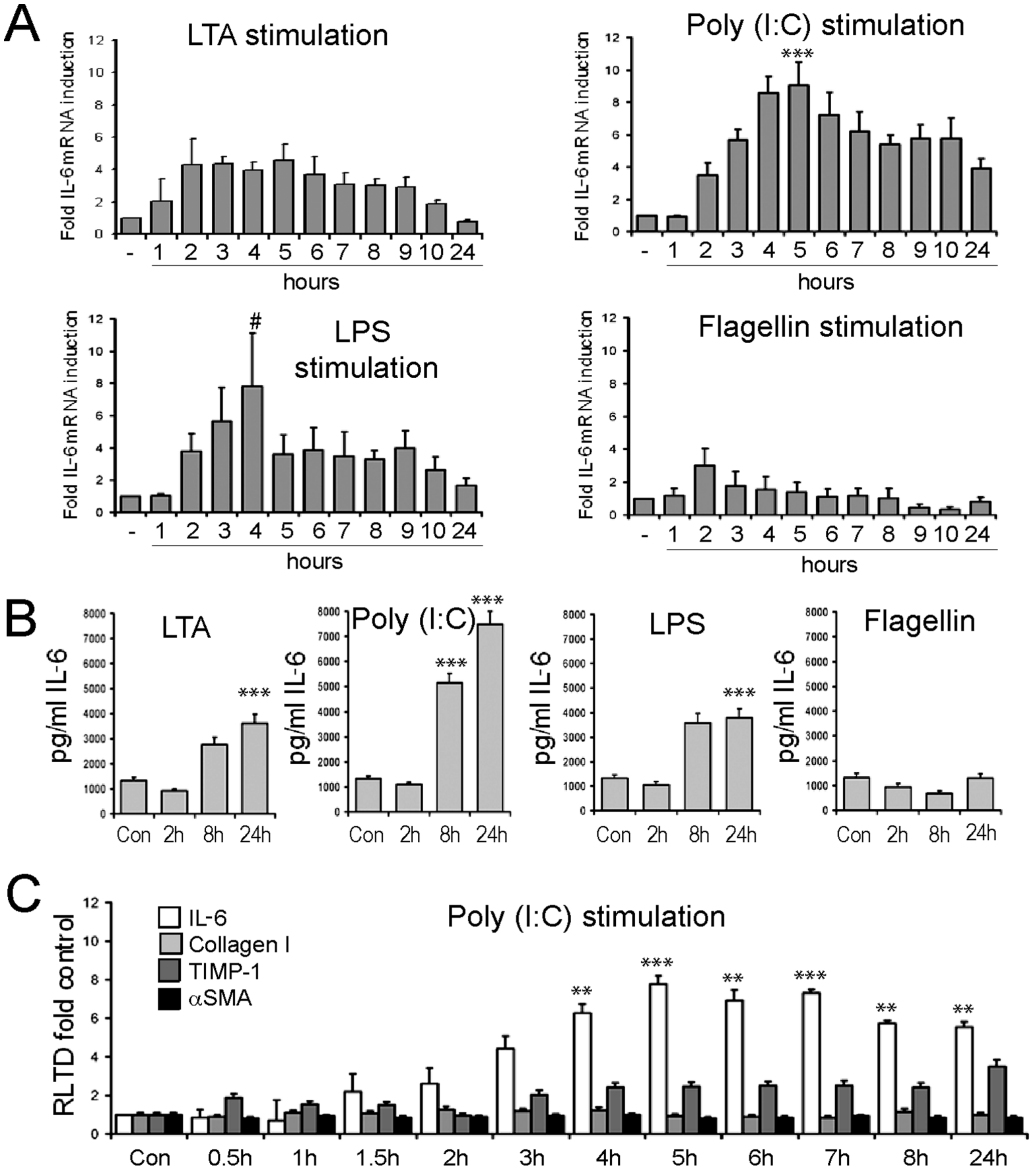
Hepatic stellate cells are responsive to stimulation by TLR ligands. (A) mRNA level of IL-6 was quantified by qRT-PCR in four separate preparations of activated rat HSCs (culture day 10) treated with TLR2 ligand (LTA, 100 ng/ml), TLR3 ligand (Poly(I∶C), 1 µg/ml), TLR4 ligand (LPS, 100 ng/ml) or TLR5 ligand (Flagellin, 1 µg/ml) for up to 24 hours (n = 4). Expression level was normalised to β actin. (B) Secreted IL-6 protein was measured by ELISA in conditioned media collected from activated HSCs treated with TLR ligands as in (A) following 2 h, 8 h or 24 h of stimulation (n = 4). (C) mRNA levels of IL-6, TIMP1, αSMA and collagen I were quantified by qRT-PCR in four separate preparations of activated rat HSCs (culture day 10) treated with TLR3 ligand (Poly(I∶C), 1 µg/ml) for up to 24 hours. Expression level was normalised to β actin. (#p<0.1, *p<0.05, **p<0.01***p<0.001).

We next determined the function of TLR3 alongside other TLRs in aHSC. Culture-activated rat HSCs were exposed between 0 and 24 hours to TLR2 (LTA), TLR3 (Poly(I∶C)), TLR4 (LPS), TLR5 (flagellin) or TLR7 (imiquimod) agonists. Measurement of IL-6 mRNA expression revealed a strong induction upon activation of TLR3 with Poly(I∶C) (9.06±1.41 RLTD, p = 0.001) and TLR4 with LPS (7.82±3.3 RLTD, p = 0.056) which peaked at 5 and 4 hours, respectively. Weaker responses were found in response to engagement of TLR2 (LTA) (4.31±1.6 p = 0.1), TLR5 (flagellin) and TLR7 (imiquimod) (data not shown) both peaking earlier at 2 hours (Figure 2A). Additionally, we monitored IL-6 secretion from aHSC in response to the same TLR agonists over a 24 hr period by ELISA (Figure 2B). LTA, LPS and imiquimod treatment was associated with a minimal 4-fold increase in secreted IL-6 at 24 hours (3622±215 pg/ml, p<0.001; 3790±64 pg/ml, p<0.001; respectively, imiquimod data not shown). Poly(I∶C) treatment resulted in a 5-fold increase in secreted IL-6 by 8 hours (5150±170 pg/ml, p<0.001) further increasing to a 7-fold induction by 24 hours (7480±28 ng/ml, p<0.001)). We saw no increase in secreted IL-6 with Flagellin treatment, confirming lack of response of aHSC to TLR5 signalling, at least in terms of IL-6 secretion (Figure 2B). We conclude that qHSC and aHSC are able to mount inflammatory cytokine responses following engagement of TLR3. However, Poly(I∶C) treatment failed to induce significantly higher levels of TIMP-1, Collagen I or α-SMA transcripts (Figure 2C), suggesting that it is unlikely that TLR3 functions as a modulator of the fibrogenic activities of HSCs.

### Activation of HSC decreases TLR3-mediated interferon response and subsequent cytokine production

**Figure 3:**
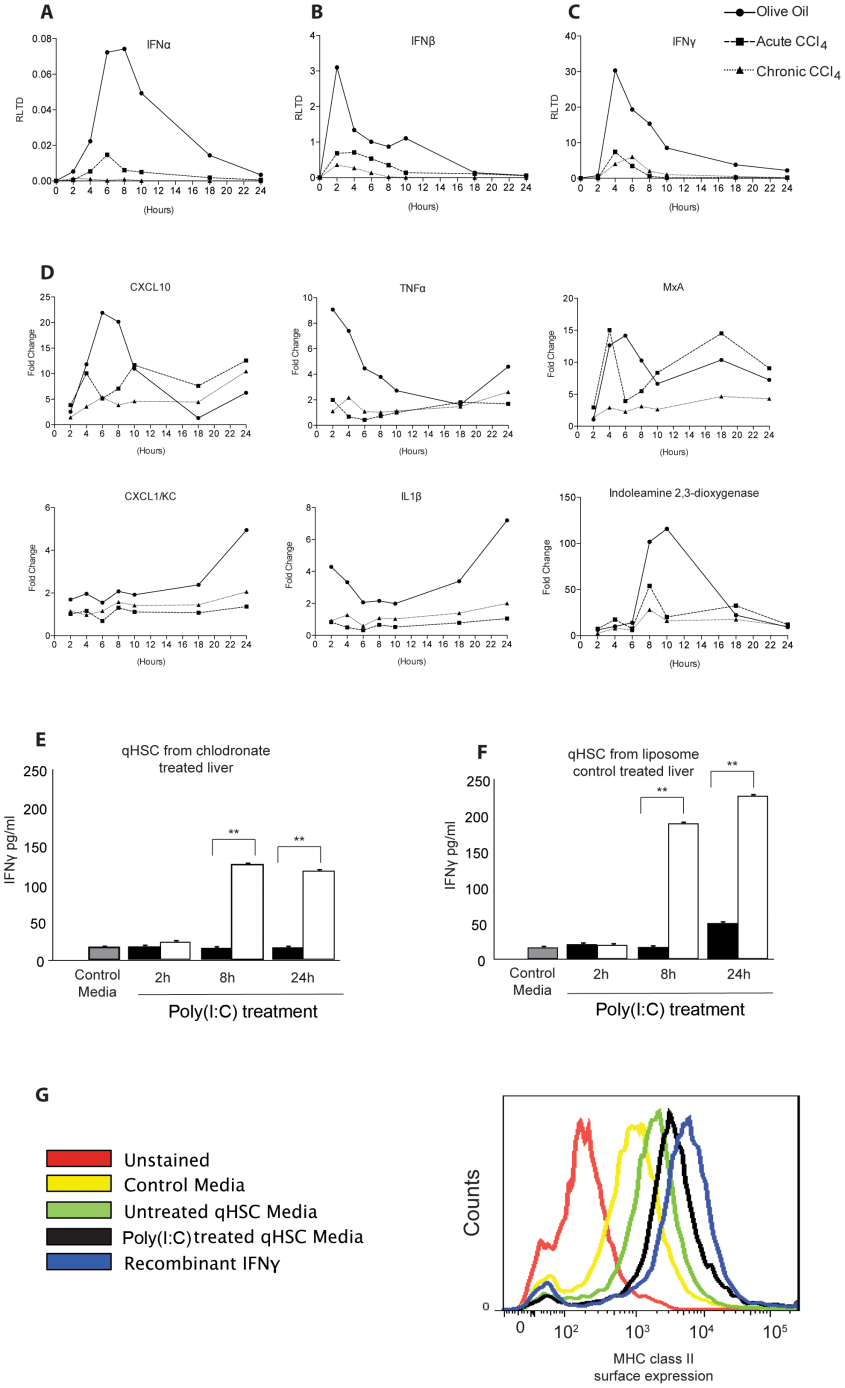
Activation of HSC decreases TLR3 mediated interferon and cytokine response. (A,B,C) HSCs were isolated from control, acute CCl_4_ treated rats (single injection), or chronic CCl_4_ treated rats (4 weeks twice weekly injections); cells were seeded onto plates and treated with Poly(I∶C) (1 µg/ml) for up to 24 hours. Interferon α, β and γ were measured and normalised to β actin. Data are expressed as RLTD. (D) Interferon-inducible cytokines CXCL10, TNF-α, MxA, CXCL1/KC, IL-1β and indoleamine 2,3-dioxygenase were measured and normalised to β actin (n = 4). Data are presented as fold change, Poly(I∶C) stimulated to unstimulated. Secreted IFNγ was measured by ELISA in the media of cultured HSCs isolated from rats treated with either chlodronate-liposomes (E) or empty liposome control (F). (G) Expression of MHC class II on macrophages cultured in qHSC conditioned media. Flow cytometric analysis of control macrophages (untreated), or macrophages incubated with control media, untreated qHSC conditioned media, Poly(I∶C) treated qHSC conditioned media or 100 ng/ml recombinant IFNγ as positive control. (*p<0.05, **p<0.01, ***p<0.001).

TLR3 plays a fundamental anti-viral role by producing interferons (IFN) in response to viral dsRNA [19]. Given that HSC express functional TLR3, we were intrigued to determine if the activation of the receptor can trigger IFN production in these cells. For this purpose we isolated HSC from 3 groups of rats; control group which was given olive oil vehicle (qHSC); rats administered CCl_4_ acutely for 48 hours (transitionary HSC) and rats receiving a chronic CCl_4_ injury for 4 weeks (myofibroblastic aHSC). HSC were subsequently treated for up to 24 hours with Poly(I∶C) prior to measurement of gene expression by qRT-PCR. HSC from untreated rats responded to Poly(I∶C) with induction of transcripts for IFNα, β and γ (Figure 3A–C). However, HSC isolated from acute and chronic CCl_4_-injured animals failed to induce IFN gene expression to similar levels seen in quiescent HSCs (Figure 3A–C). Additionally, we see failure of induction of key cytokines CXCL10, TNF-α, MxA, CXCL1/KC and IL-1β in transitionary and activated HSCs compared with quiescent HSCs (Figure 3D). Similar to Wang and colleagues, we also found indoleamine 2,3-dioxygenase was induced after stimulation of qHSCs with Poly(I∶C) [20], and this was subsequently reduced in transitionary and activated HSCs (Figure 3D). However, these findings were not universal and we found no change in induction of cytokines such as CTGF, IL-10, MCP1 and CCL5 (Figure S1).

In all studies, HSC were pre-plated in order to remove contaminating Kupffer cells (KC), however it remained possible any remaining minor contamination with KC was responsible for the observed TLR3 induction of IFN gene expression. To address this issue, HSC were isolated from rats treated with clodronate-liposomes for 48 hours to clear KC from the liver. Isolated qHSC from clodronate-treated rats were then exposed to Poly(I∶C) for 2, 8 and 24 hours prior to measurement of secreted IFNγ in the culture media. Media from control qHSC contained low levels of IFNγ, by contrast media from qHSC treated for 8 or 24 hours with Poly(I∶C) contained greater than 100 pg/ml levels of IFNγ (p<0.01 for both 8 and 24 hours)(Figure 3E). As a control for these experiments we also determined IFNγ production by qHSC isolated from rats administered carrier liposomes only (Figure 3F), these cells responded to Poly(I∶C) by producing only slightly higher levels of IFNγ than qHSC from rats exposed to clodronate-liposomes. Hence any KC contaminant in the qHSC cultures makes only a minor contribution to the overall level of TLR3-induced IFNγ in the culture model. To confirm that qHSC produce bioactive IFNγ capable of modulating immune responses, macrophages were exposed to media conditioned by qHSC exposed to Poly(I∶C) for 8 hours and induction of MHC class II was measured by FACS. Macrophage class II expression was increased in media from Poly(I∶C) treated qHSC compared with untreated media, though levels remain less than in macrophages cultured with media supplemented with maximal dose of recombinant IFNγ (Figure 3G).

**Figure 4:**
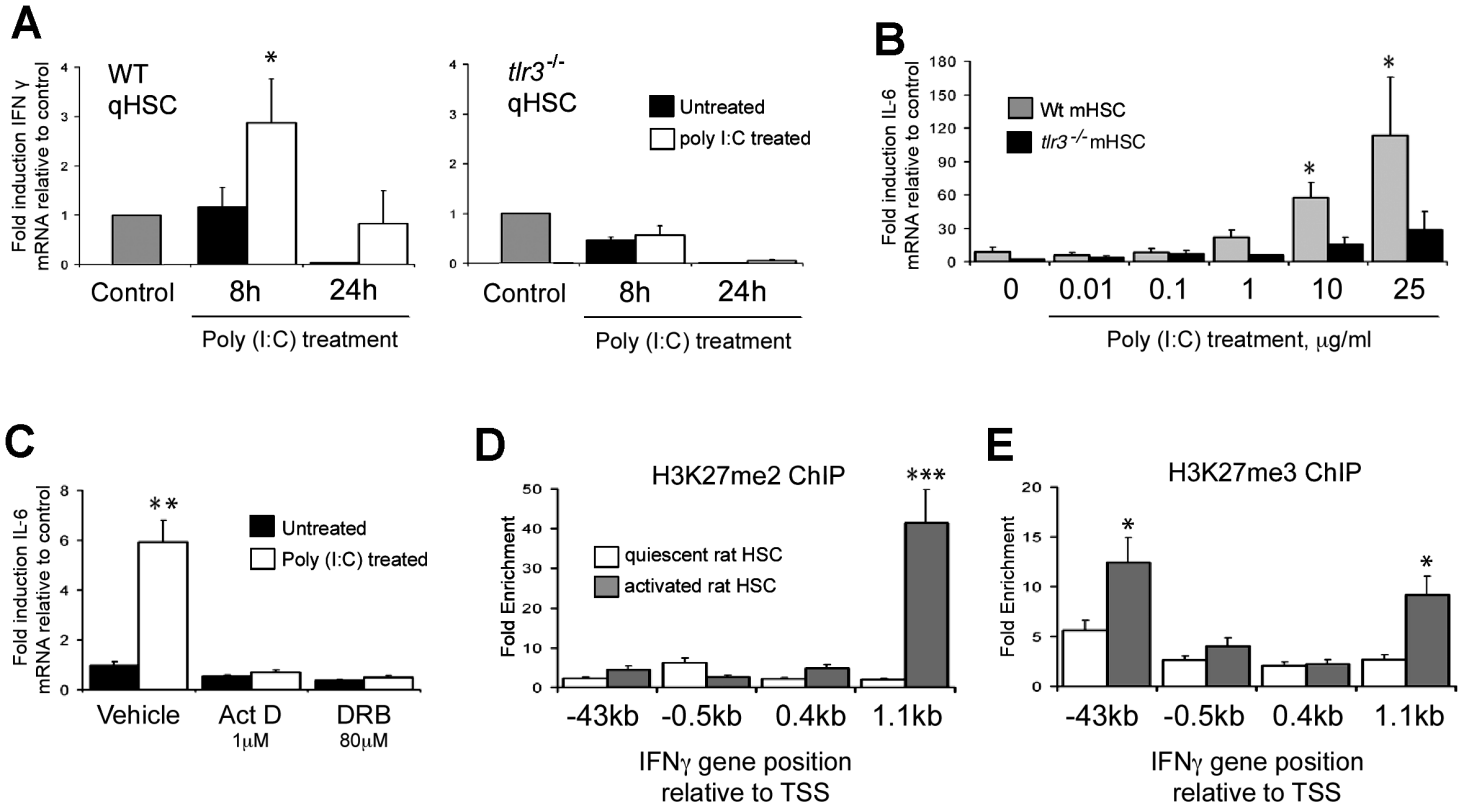
Interferon gamma and IL-6 expression are TLR3 mediated and loss of ability to induce IFNγ in aHSCs is associated with transcriptional repression by Polycomb complex. (A) WT and *tlr3* ^−/−^ activated mHSCs were treated with Poly(I∶C) (1 µg/ml) for 8 and 24 hours; subsequently IFNγ mRNA expression was measured by qPCR. Data are normalised to GAPDH and expressed as fold induction relative to control. (B) IL-6 mRNA expression was measured in WT and *tlr3* ^−/−^ activated mHSCs in response to increasing concentrations of Poly(I∶C). (C) Wild type qHSCs were treated with transcriptional inhibitors Act D (1 µM) and DRB (80 µM) for 24 hours and IL-6 mRNA expression analysed by qRT-PCR. Data are normalised to GAPDH and expressed as fold induction relative to untreated control. (D and E) One hundred micrograms of crosslinked chromatin from quiescent and activated rat HSC was incubated with 10 µg of anti-trimethyl and anti-dimethyl H3K27 antibody and ChIP assay performed. Data are expressed as fold enrichment relative to IgG control. (*p<0.05, **p<0.01, ***p<0.001).

As dsRNAs and Poly(I∶C) are able to induce innate immune responses via alternative pathways from those triggered by TLR3 (e.g. RIG-1 and MDA5) we wished to confirm that the IFN and IL-6 responses we have described can be specifically attributed to TLR3 signalling. We therefore compared responses to Poly(I∶C) between freshly isolated, quiescent wild type and *tlr3^−/−^* HSC. Treatment of wild type qHSC with Poly(I∶C) was again associated with a time-dependent induction of IFNγ which was not detected with *tlr3^−/−^* qHSC (Figure 4A) (2.83±0.8 RLTD WT control p = 0.048). For measurement of the IL-6 response, we performed a detailed Poly(I∶C) dose-response with aHSC, ranging from 10 ng/ml to 25 µg/ml (Figure 4B). For wild type aHSC, IL-6 induction was observed at 1 µg/ml (2-fold), 10 µg/ml (6-fold) and 25 µg/ml (10-fold). By contrast a modest 3-fold induction of IL-6 transcript was observed with the highest 25 µg/ml dose for *tlr3^−/−^* cells which is likely to be non-specific at this very high concentration of Poly(I∶C). Abrogation of TLR3 signalling resulted in a significant, but incomplete reduction of IL-6 mRNA expression. We cannot exclude the possibility that MDA5 or RIG-1 may play a role in this setting, however, it is worth noting a complete absence of any additional IL-6 production in *tlr3^−/−^* HSCs at 1 µg/ml of Poly(I∶C) which was the concentration used throughout all other experiments. Treatment of wild type qHSC with transcriptional inhibitors actinomycin D and 5,6-Dichlorobenzimidazole Riboside (DRB) confirmed that TLR3 mediated activation of IL-6 expression is regulated at the transcriptional level (Figure 4C). To investigate an explanation for the inability of aHSC to mount an IFNγ response following engagement of TLR3 we employed chromatin immunoprecipitation (ChIP) to determine if chromatin structure is modified at the IFNγ gene. Di- and tri-methylation of lysine 27 on Histone 3 (H3K27) is associated with transcriptionally-repressed chromatin mediated by Polycomb proteins [21]. Activated HSC acquire the repressive dimethyl-H3K27 signature within the downstream coding region of the IFNγ gene (Figure 4D). Additionally, relative to qHSC, aHSC show enrichment of trimethyl-H3K27 in both the upstream promoter and downstream coding regions (Figure 4E). These data suggest that remodelling of the HSC epigenome during their transdifferentiation to a myofibroblastic phenotype includes Polycomb-mediated silencing of the IFNγ gene.

## Discussion

The innate immune response of the liver is essential for the clearance of pathogenic microbes and initiation of the hepatic wound-healing response to liver trauma or toxic damage. TLR3 is an important component of the innate immune system providing a sensor for dsRNA originating from RNA viruses or leakage from damaged host cells [22]. In liver, TLR3 is expressed on parenchymal and non-parenchymal cells as well as infiltrating immune cells. Activation of TLR3 signalling in the liver leads to inflammation and injury through induction of NK cell activation and accumulation [23].

Augmentation of mouse liver-associated natural killer activity by biologic response modifiers occurs largely via rapid recruitment of large granular lymphocytes from the bone marrow [24]. TLR3 activated NK cells produce IFNγ which has been shown to induce apoptosis of activated HSC and to inhibit their proliferation. This in turn limits further progression of liver fibrosis [25], [26]. TLR3 is further involved in numerous processes within the liver, ranging from regeneration, viral hepatitis infection as well as autoimmune disease (reviewed in [27]).

TLR3 along with TLR4 has been implicated in the control of HCV replication via stimulation of IFNγ [28], [29]. Furthermore, TLR3 signalling has been implicated as an important controller of CD8+ T cell infiltration via the induction of IFNγ and chemokines such as CXCL9 [28]. This latter pathway has been proposed to promote liver damage and possibly facilitate the development of autoimmune hepatitis [30]. These observations suggest important but complex functions for TLR3 in liver homeostasis and immunity, as such it will be critical to define the cellular events that regulate TLR3 responses if TLR3 signalling is to be targeted therapeutically.

Non-parencymal liver cells that have previously been shown to express TLR3 in the liver include resident KC and liver sinusoidal endothelial cells (LSECs) [29], [31]. Here, we provide evidence that rodent HSC express TLR3 in both their quiescent and activated phenotypes. Of note our data are in contrast to a recently published study which reported an absence of TLR3 in quiescent HSC. The reasons for this discrepancy are unclear but may relate to differences in isolation procedures, or that while we mainly focus our studies on rat HSC, the previous study utilised mouse HSC [32]. Importantly we have also demonstrated an unexpected innate immune function for qHSC, since they can express type I and type II interferons in response to Poly(I∶C) treatment and in a TLR3-dependent manner. Interestingly this property of qHSC is lost during transdifferentiation to their activated phenotype, despite the cells retaining TLR3 expression and ability to elevate their production of IL-6 in response to Poly(I∶C) treatment. By focusing on the IFNγ gene we were able to show that aHSC acquire transcriptionally repressive chromatin modifications that may in part explain loss of IFNγ production (Figure 4D and E). Presumably this loss of IFNγ response protects aHSC from the previously documented anti-fibrogenic and pro-apoptotic actions of IFNγ, which if produced in an autocrine manner would act to suppress their fibrogenic function [33].

Recent reports suggest that the HSC phenotype is more plastic than previously thought, particularly in the context of liver injuries that may periodically resolve and recur. It is proposed that aHSC that avoid apoptosis during resolution of fibrogenesis can revert to a more quiescent phenotype (iHSC), although in a state where they are primed to activate more efficiently than naive qHSC [34], [35]. Given that iHSC may be within microenvironments where liver damage is not fully resolved, it would be interesting to determine if their phenotype reversion recovers their ability to express IFNs in response to dsRNA. Chronic production of IFNs by these cells in significant numbers and in the context of recurring liver damage may promote immune dysfunction of relevance to acute and chronic pathologies.

In summary, we have discovered an unexpected role for TLR3 in qHSC as a stimulator of type I and type II interferon expression in response to Poly(I∶C) treatment. We propose that prior to activation, qHSC may contribute to the induction of the hepatic innate immune response to injury or infection. Further investigation of the function of TLR3 on qHSC may therefore lead to strategies for modulating the recruitment and activation of innate immune cells during acute viral infections and drug-induced liver injuries.

## Supporting Information

Figure S1 **HSCs were isolated from control, acute CCl_4_ treated rats (single injection), or chronic CCl_4_ treated rats (4 weeks twice weekly injections); cells were seeded onto plates and treated with Poly(I∶C) (1 µg/ml) for up to 24 h.** We found no change in the induction of CTGF, IL10, MCP1, and RANTES (CCL5) in transitionary or activated HSCs compared with control.
(TIF)
