# Supplementary figures and images for: Quiescent Hepatic Stellate Cells Functionally Contribute to the Hepatic Innate Immune Response via TLR3
Source: PLoS One. 2014 Jan 8;9(1):e83391. doi: 10.1371/journal.pone.0083391 (PMC3885413; doi:10.1371/journal.pone.0083391)

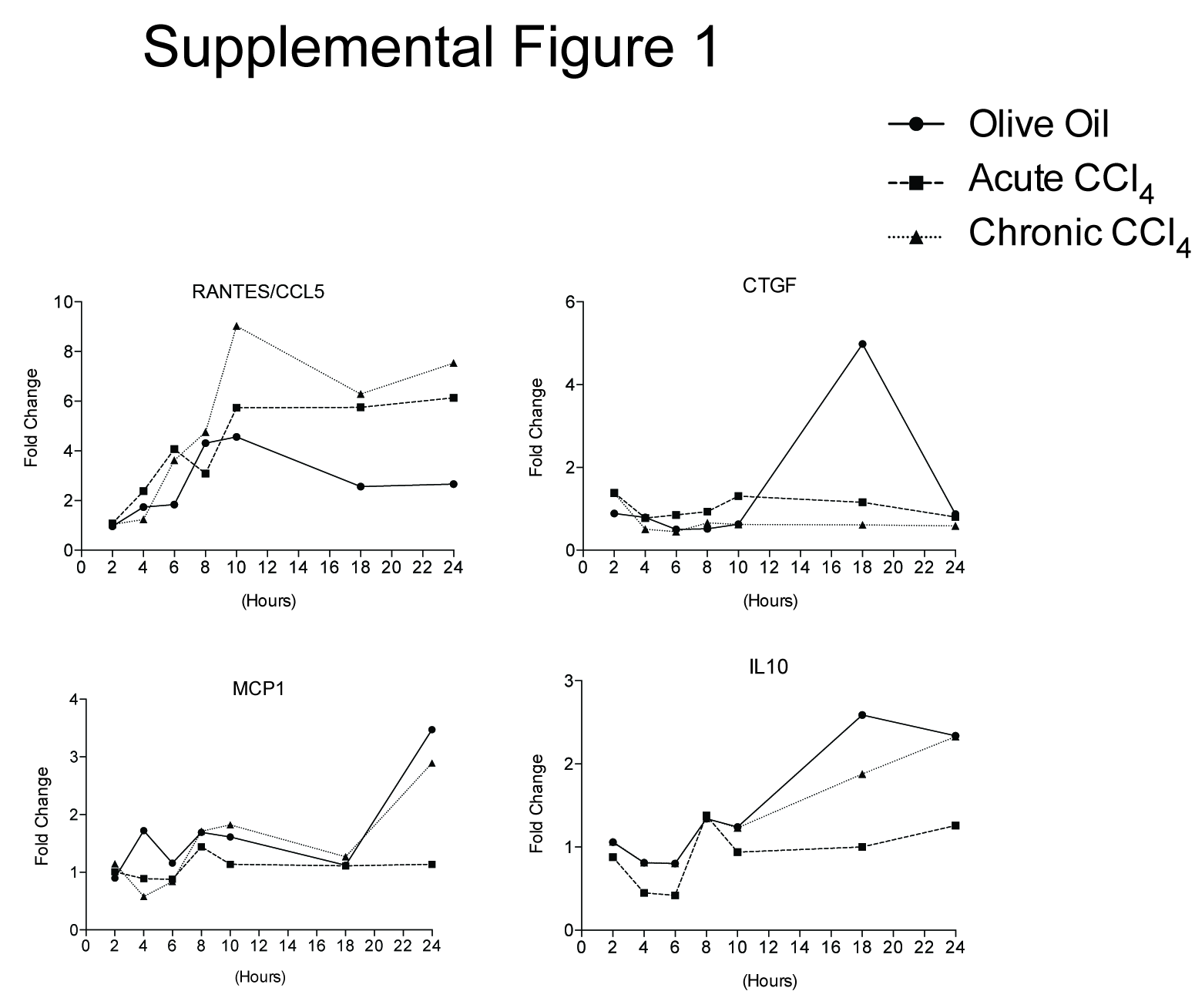

Supplement: Figure S1 — HSCs were isolated from control, acute CCl4 treated rats (single injection), or chronic CCl4 treated rats (4 weeks twice weekly injections); cells were seeded onto plates and treated with Poly(I∶C) (1 µg/ml) for up to 24 h. We found no change in the induction of CTGF, IL10, MCP1, and RANTES (CCL5) in transitionary or activated HSCs compared with control. (TIF) [file pone.0083391.s001.tif]
